# Predictors of sexual risk behaviour among adolescents from welfare institutions in Malaysia: a cross sectional study

**DOI:** 10.1186/1471-2458-14-S3-S9

**Published:** 2014-11-24

**Authors:** Nik Daliana Nik Farid, Sulaiman Che' Rus, Maznah Dahlui, Nabilla Al-Sadat, Norlaili Abdul Aziz

**Affiliations:** 1 Department of Social and Preventive Medicine, Faculty of Medicine, University of Malaya, 50603, Kuala Lumpur, Malaysia; 2 Centre for Population Health, Faculty of Medicine, University of Malaya, 50603, Kuala Lumpur, Malaysia; 3 Institute for Health Behavioural Research, Ministry of Health Malaysia, Jalan Rumah Sakit Bangsar, 50590, Kuala Lumpur, Malaysia

**Keywords:** Welfare institution, adolescents, sexual risk behaviour, smoking, family connectedness

## Abstract

**Background:**

In welfare institutions, it is essential to address the health-related needs of adolescent populations who often engage in sexual activities. This study examines the association between individual and interpersonal factors concerning sexual risk behaviour (SRB) among adolescents in welfare institutions in Malaysia.

**Methods:**

Data were derived from a cross-sectional study of 1082 adolescents in 22 welfare institutions located across Peninsular Malaysia in 2009. Using supervised self-administered questionnaires, adolescents were asked to assess their self-esteem and to complete questions on pubertal onset, substance use, family structure, family connectedness, parental monitoring, and peer pressure. SRB was measured through scoring of five items: sexual initiation, age of sexual debut, number of sexual partners, condom use, and sex with high-risk partners. Multivariate logistic regression analysis was used to examine the various predictors of sexual risk behaviour.

**Results:**

The study showed that 55.1% (95%CI = 52.0-58.2) of the total sample was observed to practice sexual risk behaviours. Smoking was the strongest predictor of SRB among male adolescents (OR = 10.3, 95%CI = 1.25-83.9). Among females, high family connectedness (OR = 3.13, 95%CI = 1.64-5.95) seemed to predict the behaviour.

**Conclusion:**

There were clear gender differences in predicting SRB. Thus, a gender-specific sexual and reproductive health intervention for institutionalised adolescents is recommended.

## Background

Sexual risk behaviour (SRB) includes premarital sex, early sexual initiation, unprotected sexual intercourse, sex with multiple partners, and unprotected sex with partners who are potential carriers of sexually transmitted infections (STIs) [1]. SRB is a major public health problem across the world, with well-documented risk factors [2]. However, the predictors of SRB among adolescents confined within welfare institutions are not well known. This gap in the literature is a cause for concern, given the linkages between SRB and adolescents' individual as well as interpersonal characteristics [3-6].

In 2011, more than 60,000 adolescents in the United States were held in residential placement facilities after breaking the law [7]. In Malaysia, the 2010 census showed a five percent reduction from the previous year in the number of new adolescents held in welfare institutions: 1096 and 1319, respectively [8]. However, this figure excluded those confined within private welfare institutions [8]. These adolescents are medically underserved and often present with significant health concerns compared with their counterparts in the community [9]. A longitudinal study conducted among 800 juvenile detainees aged 10-18 years in Chicago reported that more than 60% of adolescents had engaged in ≥10 risk behaviours, including sexual risk behaviour, at the time of their baseline interview; nearly two thirds of them persisted in ≥10 risk behaviours at the time of follow-up [10].

Adolescents in welfare institutions, including those in juvenile detention facilities, have been identified as a group that participates in sexual risk behaviours [10]. Some institutionalised adolescents may be at additional risk due to their history of substance use and lack of connectedness to family [11,12]. In addition, institutionalised adolescents have poor school attendance records and are unlikely to participate in general population surveys administered at schools. Various studies have found that these adolescents are likely to have sexual intercourse at early ages [13], have multiple sexual partners [14], and use condoms inconsistently [15,16]. These behaviours increase the risk for teenage pregnancy [17] and STIs [18,19] including human immunodeficiency virus (HIV) [20].

As with adults, adolescents detained in welfare institutions may be released at any point as they proceed through the justice system [21]. Thus, these institutions represent a unique opportunity for sexual and reproductive counselling and education, as well as screening and treatments to prevent related diseases such as STI sequelae and transmission.

This study examines the associations among individual factors (socio-demographic, self-esteem, pubertal onset, and substance use) and interpersonal factors (family structure, family connectedness, parental monitoring, and peer pressure) related to SRB among adolescents in the welfare institutions of Peninsular Malaysia. We provide recommendations for developing effective and tailored intervention programs that will improve adolescents' sexual and reproductive health within welfare institutions.

## Methods

### Study design and participants

We performed a cross-sectional study at 22 sites including children's homes, probation hostels, juvenile detention facilities, and a shelter for pregnant teenagers. These sites were located in the four regions (Northern, Central, Eastern and Southern) of Peninsular Malaysia. Study sites were selected systematically. Potential recruitment sites for adolescents were identified based on information available from the Social Welfare Department's website, along with recommendations made by the department's supervisory staff. A total of 28 sites were identified, but only 22 were approved by the department as study sites. Other institutions were excluded due to administrative issues that made data collection impossible.

All adolescents who resided in these institutions from October 2009 through June 2010 were eligible to participate in this study. Adolescents in welfare institutions are defined as persons who were younger than 18 years at the time of admission. Confinement in welfare institutions may result from a need for shelter or rehabilitation, or it may be due to abuse or unlawful activities [21]. The institutions are usually managed by a warden or principal and provide services for 10-100 individuals of a single gender category [21]. To be included in the cross-sectional study, adolescents had to meet the following criteria: 1) age ranging from 12-19 years; 2) single marital status; and 3) able to communicate in either written or spoken Malay or English language. Informed consent was obtained from the adolescents' present guardian (i.e., the Social Welfare Department of Malaysia). Additionally, participants provided verbal consent before the start of data collection.

Overall, 1082 eligible adolescents participated in the study. Participants were assured of confidentiality and the informed consent process was reviewed. The study was approved by the University Malaya Medical Centre Ethics committee.

### Study tools

Data were collected through self-administered questionnaires. All measures used in this study have been previously evaluated and found to be compatible with Malaysian adolescents. The measures are discussed below.

#### Demographic characteristics

Demographic characteristics such as age, race/ethnicity, gender, and living situation were analysed. Participants selected their ethnicity from the following options: Malay, Chinese, Indian, or Other. Participants were asked if they were living with their mother and father, mother only, father only, mother/father and new partner, or others. The variable 'others' refers to relatives, foster family, or friends.

#### Self-esteem

The Rosenberg Self Esteem (RSE) scale was used to assess adolescents' levels of self-esteem. The RSE has previously been validated among Malaysian adolescents [22]. Ten self-esteem items e.g. "On the whole, I am satisfied with myself" and "At times, I think I am no good at all" were analysed, and the subscales were scored according to standard scoring methods [23-25]. Participants' self-esteem levels were determined based on the standard scoring. In the current study, the scale was reliable, with a correlation coefficient of 0.69.

#### Pubertal onset

Puberty is marked by maturing of the genital organs, development of secondary sexual characteristics, and by the first occurrence of menstruation in the female [26]. Based on this definition, pubertal onset was assessed with an open-ended question: When did you first notice that your body was transforming, e.g. voice change in males and breast growth in females? The investigator developed this question and evaluated it prior to the actual study. The question was found to be reliable, with a Kappa value of 0.68. Based on the period when puberty normally occurs, pubertal onset was then classified as early (7-8 years old), normal (9-13 years old), or late (14-19 years old) [26].

#### Substance use

Substance use was assessed with these questions: 1) Have you ever smoked a cigarette (at least one or two puffs) or tried any tobacco products such as cigars or shisha? (Kappa = 1); 2) In the past month, did you smoke? (Kappa = 1); 3) Have you ever drunk alcohol? (Kappa = 1); and 4) Have you ever used illicit drugs? (Kappa = 1). Participants who answered 'Yes' to the questions above were categorised accordingly as smokers, alcohol drinkers, and/or illicit drug users.

#### Family connectedness

Family connectedness was assessed using measures adapted from the study 'Correlations between Family Meals and Psychosocial Well-being among Adolescents' [27]. Based on responses to the questions, 'How much do you feel you can talk to your caregiver about your problems?' and 'How much do you feel for your caregiver?' participants were categorised as experiencing low and high family connectedness. In the present study, the family connectedness items were reliable with a correlation coefficient of 0.61.

#### Parental monitoring

Parental monitoring was assessed using The Parental Monitoring Assessment (Li et al. 2000; Small and Kerns 1993), which assesses an adolescent's perception of parental monitoring on a 5-point Likert scale from 1 (never) to 5 (always) [28,29]. Respondents were classified as having a low or high parental monitoring perception. In the Malaysian setting, after adaptation and evaluation, this scale was found to be reliable, with a Cronbach alpha of 0.84. Professor Steven Small from the University of Wisconsin granted permission to use this scale.

#### Peer pressure

The Peer Pressure Scale (PPS) was used to assess whether individuals experienced pressure from peers to do certain things. This nine-item scale was previously used in an adolescent population [30]. A correlation coefficient of 0.75 was established in the present study. The PPS subscales were scored according to standard scoring methods, and norms were used to determine whether participants had reached the appropriate cut-off scores for peer pressure.

#### Sexual risk behaviour

Adolescents who had experienced sexual intercourse were further questioned regarding their sexual risk behaviours: early sexual debut, unprotected sexual intercourse, multiple sexual partners, and sex with high-risk partners (e.g., intravenous drug users). SRB items were scored to determine whether participants had reached the appropriate cut-off scores for SRB [31,32].

### Data analysis

Analyses were conducted using Predictive Analytics Software Version 17, (SPSS Inc, Chicago, IL, USA). Adolescents' socio-demographics were examined by gender. The bivariate relationships between individual and interpersonal factors and sexual risk behaviour were tested with chi-square tests. Significant associations of p < 0.05 were combined and entered into logistic regression in a single full model. The full model was run separately for each gender to test the hypothesis that the predictors of SRB would be different for males and females. Variables that were not predictive at the p < 0.05 level were deleted from the final model. Seven subjects were excluded from the analysis because of missing data.

## Results

Table 1 shows the distribution of socio-demographic characteristics of individuals in the study by gender. In terms of ethnicity, Malays were the dominant majority within the institutions (86.9%) and there were more females than males: 55.4% and 44.6%, respectively. The overall mean age of participants was 15.7 (range = 12-19). Regardless of gender, more than 50% were living with both biological parents prior to their admission into the institutions. Most of them had attended school; 75.3% and 88.6% of male and female respondents, respectively, reported having received secondary education.

**Table 1:** Socio-demographic characteristics of adolescents

Characteristics	Overall (N = 1,082) N %	Male (N = 483) N %	Female (N = 599) N %
Ethnicity*			
Malay	939 (86.9)	410 (84.9)	529 (88.5)
Chinese	38 (3.5)	25 (5.2)	13 (2.2)
Indian	92 (8.5)	46 (9.5)	46 (7.7)
Others	12 (1.1)	2 (0.4)	10 (1.7)
			
*Age group (years)*			
12-14	258 (23.8)	116 (24.0)	142 (23.7)
15-17	699 (64.6)	288 (59.6)	411 (68.6)
18-19	125 (11.6)	79 (16.4)	46 (7.7)
			
Religion*			
Islam	974 (90.1)	429 (88.8)	545 (91.1)
Buddhism	23 (2.1)	14 (2.9)	9 (1.5)
Christianity	17 (1.6)	9 (1.9)	8 (1.3)
Hinduism	59 (5.5)	26 (5.4)	33 (5.5)
Others	4 (0.4)	2 (0.4)	2 (0.3)
No religion	4 (0.4)	3 (0.6)	1 (0.2)
			
Family structure**			
Mother & Father	587 (56.0)	264 (57.0)	323 (55.2)
Mother only	211 (20.1)	97 (21.0)	114 (19.5)
Father only	55 (5.2)	18 (3.9)	37 (6.3)
Mother or father & new partner	13 (1.2)	3 (0.6)	10 (1.7)
Others	182 (17.4)	81 (17.5)	101 (17.3)
			
Highest level of education***			
Primary Education	154 (14.6)	97 (20.6)	57 (9.7)
Secondary Education	875 (82.7)	354 (75.3)	521 (88.6)
Tertiary Education	10 (0.9)	7 (1.5)	3 (0.5)

*Data is missing for 1 case. **Data is missing for 34 cases. Data is missing for 43 cases. Note: *, **, ***percentages are calculated based on less than 1082 respondents due to missing data

More than 50.0% (95%CI = 52.0-58.2) of the total sample was observed to practice sexual risk behaviours. The adolescents' reported sexual risk behaviour was examined by gender. There was a significant gender difference in terms of SRB. Female adolescents were more likely to engage in SRB compared to male adolescents (p < 0.001) (Table 2).

**Table 2:** Reported sexual risk behaviour by gender

Characteristics	Overall (N = 1082) N %	Male (N = 483) N %	Female (N = 599) N %
*Sexual debut*			
Early	458 (76.0)	162 (71.4)	296 (78.7)
Late	145 (24.0)	65 (28.6)	80 (21.3)
*No. of lifetime sexual partners*			
One partner	211 (35.6)	57 (26.5)	154 (40.8)
More than one	381 (64.4)	158 (73.5)	223 (59.2)
*Contraception use*			
Yes	520 (81.3)	211 (84.4)	309 (79.2)
No	120 (18.8)	39 (15.6)	81 (20.8)
*High-risk sexual partner*			
Yes	187 (30.2)	89 (36.9)	98 (25.9)
No	433 (69.8)	152 (63.1)	281 (74.1)
*History of STI*			
Yes	50 (8.6)	27 (12.3)	23 (6.3)
No	534 (91.4)	193 (87.7)	341 (93.7)

Note: Percentages are calculated based on number of respondents who completed the item on sexual initiation

Predictors of SRB among male adolescents included the use of substances such as tobacco, alcohol, and illicit drugs (Table 3). The findings indicated that tobacco use was the strongest factor (OR = 10.3, 95%CI = 1.22-1.80). Male adolescents who smoked tobacco were more likely to experience SRB compared to those who never smoked.

**Table 3:** Predictors of sexual risk behaviour in males

Variable	Multivariate modelling			
	B	OR	*P*	95%CI
*Tobacco use*				
Yes	2.33	1.48	<0.0001	1.22-1.80
No		1.00		Ref
*Alcohol use*				
Yes	0.92	2.50	0.004	1.35-4.63
No		1.00		Ref
*Illicit drug use*				
Yes	0.86	2.37	0.006	1.28-4.39
No		1.00		Ref

The sample size included in the logistic regression is less than the total sample of 483 due to missing data for some variables. Other variables entered: age, race, family structure, family connectedness, parental monitoring, peer pressure and pubertal onset. Hosmer-Lemeshow goodness of fit chi square = 3.53 (df = 8), p = 0.90.

Among females, three variables contributed to SRB (Table 4). Only one variable was found to be similar to the outcome of logistic regression of SRB in males: alcohol use. Compared to males, additional factors found among females were family connectedness and self-esteem. With reference to family connectedness, female adolescents with high family connectedness were more likely to engage in SRB compared to those with low connectedness.

**Table 4:** Predictors of sexual risk behaviour in females

Variable	Multivariate modelling			
	B	OR	*P*	95%CI
*Alcohol use *				
Yes	0.86	2.37	0.002	1.36-4.13
No		1.00		Ref
				
*Family connectedness*				
High	1.14	3.13	0.001	1.64-5.95
Low		1.00		Ref
				
*Self-esteem*				
Low	0.68	1.97	0.002	1.28-3.04
High		1.00		Ref

The sample size included in the logistic regression is less than the total sample of 599 due to missing data for some variables. Other variables entered: Age, race, tobacco use, illicit drug use, family structure, parental monitoring, peer pressure, pubertal onset and self-esteem. Hosmer-Lemeshow goodness of fit chi square = 8.18 (df = 8), p = 0.42.

## Discussion

The purpose of the study was to examine the associations between individual and interpersonal variables and sexual risk behaviour (SRB) among Malaysian adolescents residing in welfare institutions. The analysis showed that the majority of respondents were Malays, followed by Indians and Chinese. This ethnic distribution is almost the same as the ethnic distribution of adolescents detained for juvenile misconduct at Henry Gurney Schools and the Prison Department of Malaysia [33]. The fact that the majority of the population within public welfare institutions is Malay may explain the higher number of Malays compared to other ethnic groups.

Most of the participants were female. This can be explained by the type of illegal acts committed and abuse experienced by the adolescents placed in the selected welfare institutions. While most offenses leading to arrest are committed by boys, girls account for the majority of adolescents taken into custody for running away, prostitution, uncontrolled behaviour and teenage pregnancy [34].

A rift among family members is a contributing factor to adverse adolescent outcomes [35]. However, in this study more than half of the adolescents were living with both biological parents. This finding corresponds to a report by the Prison Department of Malaysia that found that only 19.3% of juveniles serving prison sentences come from broken homes; the remaining 80.7% have intact families [33].

The use of substances such as cigarettes and illicit drugs was associated with SRB among males. These findings are consistent with previous studies that found that cigarette smoking and SRB are correlated with each other [36,37]. This could be due to conduct problems among institutionalised adolescents. A study of Greek adolescents found that conduct problems are associated with adolescent smoking [38]. Cigarette smoking, which led to conduct problems, in turn influenced adolescents to engage in SRB. Previous research has revealed that children with high rates of aggressive disruptive behaviours and attention problems at school entry are more likely to engage in problem behaviours in middle school; these behaviours are associated with early initiation of sexual activity [39].

Other studies have also found that adolescents who use illicit drugs may show a tendency to engage in SRB [11,39]. This association can be explained by high levels of sensation seeking [40]. High levels of sensation seeking have been directly linked to increased likelihood of drug use, which has been shown to influence SRB among boys [41-43]. Previous studies have shown that institutionalised adolescents are more likely to adopt high-risk behaviour such as substance use [9].

The current study identified alcohol use as a predictor of SRB for both male and female adolescents. This finding is consistent with previous studies that linked alcohol use to SRB [44,45]. To adolescents, alcohol is commonly used as a way of socialising with peers and coping with stress. Alcohol also facilitates contact with the opposite sex. This behaviour will eventually increase adolescents' involvement in SRB.

High family connectedness and low self-esteem were noteworthy factors of SRB among females. The current study found that female adolescents with high family connectedness were more likely to engage in SRB. This result contradicts findings from a previous review of studies of the general adolescent population. The review indicated that lower family connectedness was associated with sexual risk behaviour and higher connectedness was a protective factor [46]. The distinctive result found in the current study is probably due to mitigating circumstances. Although female adolescents who engaged in SRB might have had a perception of good family connectedness, parental monitoring might have been compromised. Adolescents' behaviour in front of their caregivers is often controlled, giving their parents the impression of obedience. This controlled behaviour fosters a better relationship between parent and child after institutionalisation.

Female adolescents with low self-esteem were more likely to engage in SRB. This finding is in contrast to a study that reported no significant association between self-esteem and SRB in female adolescents [47]. However, several studies have identified low self-esteem as a determinant of SRB [48,49]. Studies in a wide range of western countries have determined that adolescent females, on average, have lower self-esteem compared to males [50,51]. A previous study of self-esteem among Malaysian adolescents found that female adolescents are more likely than males to have low self-esteem [52]. In the current study, the adolescents' low self-esteem could be due to a lack of consistent communication with, support from, and encouragement by parents and other adult role models. Adolescents with low self-esteem are more likely to succumb to pressure from peers who practice SRB. Low self-esteem can make adolescents more vulnerable to SRB if they feel the need to experience SRB in order to fit in with their peers.

### Strengths and limitations

The following limitations should be noted when interpreting some of the findings. Due to the cross-sectional nature of the study, the findings offer only a snapshot of the adolescents' SRB. Also, if one intends to look at causal relationships between the factors and sexual risk behaviour, temporal relationships between those factors should be examined carefully. Nevertheless, indication of associations was useful in generating hypotheses for future research. Even though the population sampled in this study was limited to adolescents from welfare institutions, this population could be said to represent at-risk adolescents in Malaysia.

### Recommendation

The relationship between substance use and SRB illustrates the importance of addressing substance use when aiming to reduce adolescents' SRB. In order to change adolescents' attitudes towards substance use, individual as well as environmental interventions are required. It is necessary to develop interventions and programs for institutionalised adolescents that address substance use and its consequences for sexual and reproductive health. It is also important to involve the family in early religious and moral education (that is, education based on religion and culture). Another approach to be considered is a stricter policy prohibiting underage individuals from substance use in venues such as discos, concerts and bars. Targeted programs at these venues can promote behaviour that is more responsible and educate adolescents on the consequences of smoking cigarettes, drinking alcohol and using illicit drugs.

The link between family connectedness, self-esteem and SRB among female adolescents indicates the need for better individual and interpersonal prevention programs. Although female adolescents with SRB perceived high levels of family connectedness, other factors such as parental monitoring were probably lacking. Caregivers and teachers should be educated about and fully understand the risks associated with not monitoring adolescents. Parental monitoring can help protect adolescents from potential problems caused by the use of internet and social media outlets. Interventions and health counselling should be tailored to help adolescents with low self-esteem. In general, interventions should involve building adolescents' skills in areas in which they are deficient. Teachers and community members can carry out these interventions. Family members and teachers can also prevent or reduce low self-esteem by reducing social comparison cues and offering encouragement.

In the course of this study, more questions about SRB have emerged. Future research should use other data collection methods such as in-depth interviews. To our knowledge, there have been no previous longitudinal studies of sexual risk behaviour in the Malaysian context. Longitudinal studies can help to unravel developmental progressions, identifying which factors come into play earlier and which come later in the development of SRB.

Adolescents in welfare institutions need to be aware of how to protect themselves from the consequences of SRB. Interventions should convey the message that anyone who is or will be sexually active is at risk for contracting sexually transmitted infections and experiencing teenage pregnancy.

## Conclusions

This cross-sectional study shows that SRB among male adolescents is determined predominantly by individual factors such as substance use. For most female adolescents, SRB was linked to both individual and interpersonal factors such as self-esteem and family connectedness. These findings indicate the need for gender-specific interventions that aim to reduce SRB by preventing adolescent substance use, increasing adolescent self-esteem, improving adolescents' life skills, and promoting good parenting skills.

## Competing interests

The authors declare that they have no competing interests.

## Authors' contributions

ND, SCR, MD, NAS and NAA conceived the study. ND, MD and NAS drafted the manuscript. ND and SCR undertook the statistical analysis. All of the authors read and approved the final manuscript.
